# Differences between Facebook and Instagram Usage in Regard to Problematic Use and Well-Being

**DOI:** 10.1007/s41347-021-00229-z

**Published:** 2021-10-13

**Authors:** Maria Limniou, Yasmin Ascroft, Sarah McLean

**Affiliations:** Department of Psychology, University of Liverpool, Liverpool, UK

**Keywords:** Facebook, Instagram, Self-esteem, Depression, Loneliness, Problematic use

## Abstract

Although the association of well-being (e.g., self-esteem, depression symptoms, and loneliness) with Facebook usage (i.e., number of friends and frequency of use) has been investigated by many researchers, only a limited number of studies have scrutinised Facebook against Instagram use. The aim of this study is not only to address this literature gap but also to explore whether problematic use and well-being differ between Facebook and Instagram users in relation to the number of received “likes” and Facebook friends/Instagram followers, as well as the importance of these “likes” and friends/followers. Sixty-nine Facebook users and 66 Instagram users completed an online questionnaire, including self-esteem, loneliness, depression, and problematic Internet use items. Overall, Instagram users exhibited significantly higher problematic use behaviour compared to Facebook users. Age and importance of “followers” were negatively associated with problematic use of Instagram, whilst only the importance of “likes” was negatively associated with Facebook. The number of Facebook “friends” was positively associated with depressive symptoms, but this was not the case for the number of Instagram “followers”. It is important to note that the number of “likes” was only negatively associated with self-esteem, but there was no association with loneliness. A potential explanation between the differences in the two platforms and well-being might be related to their different functionalities, for example, Instagram is an image-oriented platform that may boost users’ self-esteem through post “likes” but only when the users are strongly connected.

## Introduction

Facebook, a social networking site, was launched in 2004. It allows users to be connected online with “friends” (i.e., family members, real friends, colleagues, and/or people who they do not know), exchange ideas, and share resources (i.e., videos, pictures, music). The Facebook “like” feature allows users to interact with their “friend’s” posts. By receiving Facebook “likes”, users feel that they are supported by their online “friends” (Ellison et al., 2014; Wohn et al., 2016). The popularity of Facebook has attracted pivotal research into the nature of its use in relation to individuals’ well-being, such as self-esteem, emotional support, and social connection. This has occurred on small scales among young populations: for example, a study involving 70 undergraduate students found that those with low self-esteem spent a lot of time on Facebook (Kalpidou et al., 2011). Research has also been conducted on large scales, such as the study by Andreassen et al. (2017), in which the addictive social media use of 23,532 Norwegians from all ages was connected to low self-esteem. Bergagna and Tazghini (2018) have mentioned that individuals with low self-esteem are interested in social comparison (the difference between individuals’ abilities and personal characteristics), spending a great amount of time on Facebook. Furthermore, the number of “likes”, users’ profile status, and/or photos of their Facebook “friends” may support social comparisons due to the speed and ease with which individuals can connect to their peers (Steers et al., 2014). Many researchers have discussed how the greater tendency towards social comparison might increase Facebook use, motivated by the search for information about others, influencing self-esteem and depression (Nesi & Prinstein, 2015; Tartaglia, 2016). McCloskey et al. (2015) found that minimal “likes” and Facebook “friends” were predictive of greater depressive symptoms due to a lack of emotional support users received. Additionally, Phu and Gow (2019) found that, although persistent Facebook usage is associated with high loneliness levels, the number of Facebook “friends” is highly associated with subjective happiness, due to emotional connectedness. Facebook users might gain acceptance amongst their peers and reconstruct social networks by facilitating their needs of belongingness to an online community (Beyens et al., 2016). This could decrease depressive symptoms by increasing users’ feelings of self-worth (Nesi & Prinstein, 2015), which may be connected to the sociometer theory (self-esteem is directly related to social relations and interactions monitoring the acceptance and/or rejection from others) (Stefanita et al., 2018). However, researchers who have studied the role of social media (mainly Facebook) on users’ well-being, self-esteem, depression symptoms, and anxiety have presented mixed findings regarding the significant association with a potential explanation based on passive or active use (Escobar-Viera et al., 2020). A recent study regarding social support and feelings of connectedness over the COVID-19 pandemic has shown the passive usage of Facebook is negatively related to well-being through upward social comparison, whereas active usage of Instagram is positively related to satisfaction with life and negative affect through social support (Masciantonio et al., 2021).

Instagram, another social media platform, was launched in 2010. It is more image focus-oriented than Facebook, allowing users to enhance the appearance of their photos through several filters. Instagram users can share their own created images with their private (“followers”) or with the wider public network, allowing other Instagram users to “like” their photo posts. Users can also “tag” their images with identifying words, making them easy to search for themed content. Similar to the studies conducted on Facebook, research has explored the role of Instagram on psychological well-being (e.g., self-esteem, depression symptoms, and loneliness) (Mackson et al., 2019). For example, Yang (2016) studied the role of loneliness, social comparison orientation, and Instagram usage, where Instagram broadcasting was positively correlated with loneliness and Instagram interaction was associated with lower loneliness only for low social comparison orientation users. Additionally, Lup et al. (2015) found the association between Instagram use and increased depressive symptoms, when users followed fewer “strangers” online. These findings could be explained due to users following seemingly more attractive peers, leading them to engage in upward comparisons, which might increase the severity of depressive symptoms. Martinez-Pecino and Garcia-Gavilán (2019) explored the influence of “likes” and self-esteem on problematic Instagram use (excessive engagement in social media) of teenagers, revealing that the problematic use of “likes” had a low impact on individuals with high self-esteem.

Problematic social media use (PSMU) has been explored by many researchers. It has been described as the “negative outcomes triggered by the excessive use of social media which may have a detrimental effect on the personal, social, and/or professional lives of the users” (Banyai et al., 2017, p. 2). Recent studies have explored how well-being affects social needs and problematic Instagram use (Kırcaburun & Griffiths, 2019; Ponnusamy et al., 2020), whilst most older studies have been mainly focused on Facebook problematic use and well-being. For example, research has shown that problematic Facebook use is associated with low self-esteem, due to increased social comparison opportunities (Marino et al., 2018; Vogel et al., 2014); increased depressive symptoms, if their peers appeared to have more fulfilling lives than their own (Shensa et al., 2017); and increased loneliness (Błachnio et al., 2016; Błachnio & Przepirorka, 2019). Problematic Facebook users reported three times the rate of perceived social isolation, increased odds of depressive symptoms, and reduced self-esteem, compared to irregular users (Chen & Lee, 2013; Primack et al, 2017).

A recent study has found that there was an association between Instagram use frequency and problematic use, but not with depression and anxiety (Rozgonjuk et al., 2020). Social comparisons may lead Instagram users to feel unfulfilled in their own lives, increasing their depressive symptoms (Stapleton et al., 2017). However, only limited studies have explored the role of Instagram use on individuals’ well-being, such as self-esteem, depression symptoms, and loneliness, compared to Facebook (Ponnusamy et al., 2020). Thus, the current study aims to investigate the association between problematic Instagram and Facebook use in relation to self-esteem, depressive symptoms, and loneliness, in order to understand whether either platform is highly associated with negative psychological well-being. Specifically, the objectives of this study are to explore:

- the difference between PSMU, depressive symptoms, loneliness, and self-esteem between Facebook and Instagram users;
- the association between PSMU for each social media platform with depressive symptoms, loneliness, and self-esteem;
- the association between Facebook “friends”, Instagram “followers”, and social media “likes” with depressive symptoms and loneliness, and self-esteem;
- the difference between PSMU, depressive symptoms, loneliness, and self-esteem between and within Facebook and Instagram users after controlling the participant age; and
- the association between age, the number of “likes”, and the importance of “likes”, along with either Facebook “friends” and the importance of Facebook “friends” or Instagram “followers” and the importance of Instagram “followers” with PSMU.

The overall hypothesis of this study is that Instagram and its features have a greater impact on individuals’ well-being compared to Facebook. This hypothesis is supported by social comparison theory (Festinger, 1954) and the fact that Instagram is a highly photo-oriented social media platform, exposing users to more images than Facebook, causing them to seek out comparisons with their Instagram “followers”. In order to address the above aim and the hypothesis of this study, the same questionnaire over the same period of time was distributed to Facebook and Instagram users. There is not a similar research study that the authors are aware of which explores the same well-being factors for Facebook and Instagram using the same questionnaire for both social media platforms users over the same period of time.

## Method

### Participants and Experimental Conditions

An ethical approval about this research study has been initially gained from the University Ethics Committee. Before participating in this study, a participant information sheet and a consent form were provided to participants. The participant information sheet included details about this study, such as the aim, the reason that these participants have been invited and the inclusion criteria (i.e., have a Facebook/Instagram account, older than 18 years old, and individuals who have been clinically diagnosed with depressive symptoms). Participants were also informed that they could anonymously withdraw at any time without providing any reason, if they wished to do so. After providing their consent, participants could anonymously complete the online questionnaire, which was the same for both Facebook and Instagram users.

This is an opportunity sampling research design; the online questionnaire was advertised on the researchers’ social media networks and the School of Psychology recruitment website, on which first-year psychology undergraduate students gain research experience through their participation in research studies. This recruitment process ensures that the study can reach participants of various ages.

Overall, 135 social media users fully completed the online questionnaire over a two-month period (December 2019–January 2020). Based on power calculation, the margin error of the 135 participants who completed the questionnaire was ± 7.2% for a total population of 500 social media users. At the beginning of the questionnaire, the participants were asked whether they mostly were Facebook or Instagram users, based on the one that they spend more than 75% of their time on. Based on participants’ responses 66 (48.9%) were mostly Instagram users, whilst 69 (51.1%) were mostly Facebook users. Seventy-nine (58.5%) participants were aged between 18 and 30, and 56 (41.5%) participants were aged 31 and over. Forty-three (31.9%) males and 92 females (68.1%) participated in this study. Table 1 further illustrates the gender and age split of participants’ preference on using either Facebook or Instagram.

**Table 1:** Demographics (age and gender) by preferred social media platform

	Facebook (%)	Instagram
Male	44%	20%
Female	56%	80%
Age between 18 and 30	26%	82%
Age 31 and over	74%	18%

### Questionnaire

The online questionnaire included 66 items and was designed using Qualtrics (www.qualtrics.com). The initial part of the questionnaire included 10 items; participants were also asked about the subjective importance of friends and followers and their motivation for utilising Facebook or Instagram. Specifically, this part gathered demographic information (gender and age), social media usage (numbers of “likes”, number of Facebook “friends” and Instagram “followers”, and their importance to them), and an item regarding the main reasons of using social media. These were multiple-choice items, and the approximately 15–20 min-long questionnaire is located at the ZENODO repository (https://zenodo.org/record/5017900#.YNMw7kzTVPY).

In the next part of the questionnaire, validated items about individuals’ personal feelings of social media connection to their psychological well-being (e.g., self-esteem, loneliness, and depressive symptoms) were included. Specifically, the Rosenberg Self-Esteem Scale (Rosenberg, 1965) consisted of 10 items that assessed both positive and negative feelings about the self. Five items contained positively worded questions about the self (e.g., “I am able to do things as well as most other people”). The remaining 5 items contain negatively worded questions about the self (e.g., “I certainly feel useless as times”). Responses were recorded utilizing a 4-point Likert scale, from 0 (strongly disagree) to 3 (strongly agree). The total self-esteem scores ranged from 0 to 30, where higher scores were emblematic of high self-esteem. The Cronbach’s alpha was 0.84.

The revised 20 items UCLA loneliness scale (Russell et al., 1978) was used to measure participants’ subjective feelings of loneliness and social isolation. This scale was both positively worded (e.g., “I feel in tune with the people around me”), reflecting satisfaction with social relationships for several questions, and negatively worded (e.g., “I am no longer close to anyone”), illustrating dissatisfaction with such relationships in the remaining. Responses were recorded utilizing a 4-point Likert scale from 1 (never) to 4 (often). The total loneliness scores ranged from 20 to 80, where high scores were indicative of high loneliness. The Cronbach’s alpha was 0.91.

The Center for Epidemiological Studies Depression Scale (CES-D) (Radloff, 1977) consisted of 20 items and was used to measure how often over the past week participants experienced feelings associated with depression (i.e., poor appetite and restless sleep). The scale included 4 positively worded items (e.g., “I was happy”) and 16 negatively worded items (e.g., “people were unfriendly”). Responses were recorded utilizing a 4-point Likert scale from 0 (rarely/none of the time), to 3 (most/all of the time). The total depression scores ranged from 0–60, with the higher scores being indicative of greater depressive symptoms. The Cronbach’s alpha was 0.82.

The modified Bergen Social Media Addiction Scale (BSMAS) (Andreassen et al., 2017) of the previously validated Bergen Facebook Addiction Scale (BFAS; Andreassen et al., 2012) was used to measure problematic social media use (PSMU). In this modified scale, the words “social media” were used instead of the word “Facebook”, with social media being defined as “Facebook or Instagram” in the instructions, depending on what was mostly used by the users. The Bergen Social Media Addiction Scale (BSMAS) was used to assess social media use in general over the past 12 months. This 6-item scale has been developed based on social media addiction according to six basic addiction symptoms noted earlier (i.e., salience, conflict, mood modification, withdrawal, tolerance, and relapse) (Griffiths, 2005) asking individuals to rate their experiences occurring over the past year (e.g., “How often during the last year have you become restless or troubled if you have been prohibited from using social media?”), measured against a 5-point Likert scale (1 very rarely to 5 always). The total score of BSMAS ranges from 6 to 30, with the higher scores being indicative of greater addiction indicators. The Cronbach’s alpha was 0.85.

## Results

In relation to the descriptive question regarding the main reasons for using Facebook or Instagram, both groups mentioned their needs for communication, keeping themselves informed by reading posts, or following links and events. However, a significant difference has been identified in Instagram users’ responses, as they exhibited behaviours such as looking at others’ photos and profiles, keeping everyone updated with what they are doing, and looking at photos to pass their time (Table 2).

**Table 2:** A chi-square analysis on participants’ responses about the main reasons for using either Facebook or Instagram

Main reason	Facebook (%)	Instagram (%)	Chi-square
I enjoy looking at different pictures on social media	45%	85%	*χ*^2^(1, 135) = 23.462, *p* < 0.05
I like to keep everyone updated with what I am doing	11%	32%	*χ*^2^(1, 135) = 8.180, *p* < 0.05
As a way of passing the time	52%	74%	*χ*^2^(1, 135) = 7.045, *p* < 0.05
To find funny content	48%	70%	*χ*^2^(1, 135) = 6.647, *p* < 0.05
To check other profiles	20%	39%	*χ*^2^(1, 135) = 5.905, *p* < 0.05

*α* (0.05) is the limit of significance level, *χ*^2^(a, b) is the variance between groups, *p* is significance level

### Difference Between Social Media Users with PSMU, Self-esteem, Loneliness, and Depression

Table 3 presents the descriptive values for Facebook and Instagram users, regarding to their depressive symptoms, loneliness, self-esteem, and Problematic Social Media Use (PSMU), along with the one-way ANOVA statistical analysis findings. Overall, there was a significant main effect of social media platform on PSMU, with Instagram users having significantly higher PSMU scores than Facebook users. However, there was no significant difference between Facebook and Instagram users in depressive symptoms, loneliness, and self-esteem. A MANOVA statistical analysis also revealed that there was a significant overall effect of social media platform upon all the dependent variables, *F*(4, 130) = 2.87, *p* = 0.026, *np*2 = 0.08.

**Table 3:** Descriptive statistics for Facebook and Instagram users along with the ANOVA statistical analysis per scale

Dependent variable	Facebook user *M* (± SD)	Instagram user *M* (± SD)	ANOVA between social media users (*α* = 0.05)
Self-esteem	15.2 (± 3.52)	14.8 (± 3.37)	*F*(1, 33) = 0.36, *p* = 0.550, *np*2 = 0.00
Loneliness	39.4 (± 11.67)	37.2 (± 11.02)	*F*(1, 33) = 1.34, *p* = 0.250, *np*2 = 0.01
Depressive symptoms	30.6 (± 9.58)	31.3 (± 8.87)	*F*(1, 33) = 0.18, *p* = 0.670, *np*2 = 0.00
PSMU	11.4 (± 3.84)	13.4 (± 4.18)	*F*(1, 133) = 6.59, *p* < 0.01, *np*2 = 0.05

*α* the limit of the significant level, *M* mean, *SD* standard deviation, *F(a,b)* is the variance value, *p* significant value, *np*^*2*^ size effect

### PSMU for Each Social Media Platform with Depressive Symptoms, Loneliness, and Self-esteem

Multiple regression analyses^1^ were conducted, to explore whether the PSMU for each social media platform was associated with depressive symptoms, loneliness, and self-esteem. The regression model predicted approximately 9.7% of the overall variance in PSMU for Facebook users, *ΔR*2 = 0.137, *F*(3, 65) = 3.445, *p* = 0.02. However, there was no significant association between PSMU and loneliness (*β* = 0.05, *p* = 0.39), depressive symptoms (*β* = 0.09, *p* = 0.14), and self-esteem (*β* = 0.09, *p* = 0.47).

The regression model predicted approximately 0% of the overall variance in total PSMU for Instagram users, *ΔR*2 = 0.05, *F*(3, 62) = 1.02, *p* = 0.34. There was no significant association between PSMU and loneliness (*β* =  −0.07, *p* = 0.66), depressive symptoms (*β* = 0.15, *p* = 0.13), and self-esteem, (*β* = 0.07, *p* = 0.66).

### Facebook “Friends”, Instagram “Followers”, and Social Media “Likes” with Depressive Symptoms and Loneliness, and Self-esteem

Table 4 illustrates participants’ responses regarding Facebook and Instagram “likes” and “friends/followers”.

**Table 4:** Participants’ responses per social networking site (Facebook or Instagram) on questions regarding the numbers of “likes” and the number of friends/followers

	Facebook users	Instagram users
Number of “likes” which users often receive on their social media account		
0–60	81%	26%
61–120	20%	33%
121–180	0%	17%
181 and above	0%	24%
Number of “friends/followers” which users have on their social media		
0–60	13%	0%
61–120	13%	9%
121–180	12%	8%
181–300	20%	17%
301 and above	42%	67%

A multiple regression was conducted to investigate whether the number of Facebook “friends” and Instagram “followers” and “likes” are associated with depressive symptoms. The regression model predicted 6% of the variance in overall depressive symptoms scores, *ΔR*2 = 0.06, *F*(3, 67) = 2.44, *p* = 0.072. The number of Facebook “friends” was positively associated with depressive symptoms (*β* = 0.28, *p* < 0.030). However, neither Instagram followers (*β* =  −0.06, *P* = 0.734), nor likes (*β* =  −0.19, *p* = 0.211), were significant predictors of depressive symptoms.

Another multiple regression was conducted to explore whether the number of Facebook “friends” and Instagram “followers” and “likes” are associated with loneliness. The regression model predicted 6% of the variance in overall loneliness scores, *ΔR*2 = 0.06, *F*(3,67) = 2.46, *p* = 0.070. Neither Facebook friends (*β* = 0.10, *p* = 0.432), Instagram followers (*β* =  −0.26, *p* = 0.119), nor likes (*β* =  −0.12, *p* = 0.478) were significant predictors of loneliness.

A multiple regression was conducted to investigate whether the number of Facebook “friends” and Instagram “followers” and “likes” are associated with self-esteem. The regression model predicted 5% of the variance in self-esteem scores, *ΔR*2 = 0.05, *F*(3,67) = 2.14, *p* = 0.103. There was a significant negative association between “likes” and self-esteem (*β* =  −0.36, *p* < 0.023). However, neither Facebook “friends” (*β* = 0.10, *p* = 0.429) nor Instagram “followers” (*β* =  −0.15, *p* = 0.354) were significant predictors of self-esteem.

### Difference Between PSMU, Depressive Symptoms, Loneliness, and Self-esteem Between and Within Facebook and Instagram Users After Controlling the Participant Age

A one-way ANCOVA statistical analysis was conducted to compare psychological well-being factors and PSMU between the two platforms (Instagram and Facebook) after controlling users’ age (Table 5). There was not any significant difference between Facebook and Instagram users, and similarly, it was the case after conducting a one-way ANOVA statistical analysis to compare the psychological well-being factors within each social media group (Facebook and Instagram users).

**Table 5:** Comparisons between the participants’ responses per social media platform related to self-esteem, loneliness, depressive symptoms, and PSMU after controlling participants’ age belonging to one of the two groups

**Age groups**	**Social media (*M*, SD) per each age group**	**One-way ANCOVA between and ANOVA within age groups (*α* = 0.05)**
Self-esteem	Facebook: 14.3 (± 2.97) Instagram: 14.8 (± 3.42)	*F*(1, 132) = 0.118, *p* = 0.73, *η*^2^ = 0.001 Within Facebook participants: *F*(1, 67) = 1.395, *p* = 0.24, *η*^2^ = 0.02 Within Instagram participants: *F*(1, 64) = 0.069, *p* = 0.79, *η*^2^ = 0.001
18–30 years old		
Above 31 years old	Facebook: 15.5 (± 3.68) Instagram: 15.2 (± 2.86)	
Loneliness	Facebook: 39.6 (± 8.99) Instagram: 36.9 (± 10.91) Facebook: 39.4 (± 12.48) Instagram: 40.4 (± 13.28)	*F*(1, 132) = 0.432, *p* = 0.51, *η*^2^ = 0.003 Within Facebook participants: *F*(1, 67) = 0.003, *p* = 0.96, *η*^2^ = 0.00 Within Instagram participants: *F*(1, 64) = 0.457, *p* = 0.50, *η*^2^ = 0.07
18–30 years old		
Above 31 years old		
Depressive symptoms	Facebook: 35.6 (± 7.07) Instagram: 31.5 (± 8.83) Facebook: 29.9 (± 10.29) Instagram: 31.3 (± 8.87)	*F*(1, 132) = 0.232, *p* = 0.63, *η*^2^ = 0.002 Within Facebook participants: *F*(1, 67) = 0.992, *p* = 0.32, *η*^2^ = 0.02 Within Instagram participants: *F*(1, 64) = 0.361, *p* = 0.55, *η*^2^ = 0.01
18–30 years old		
Above 31 years old		
PSMU	Facebook: 12.1 (± 3.46) Instagram: 13.4 (± 4.22) Facebook: 11.1 (± 3.97) Instagram: 9.8 (± 1.30)	*F*(1, 132) = 0.504, *p* = 0.48, *η*^2^ = 0.004 Within Facebook participants: *F*(1, 67) = 0.924, *p* = 0.34, *η*^2^ = 0.014 Within Instagram participants: *F*(1, 64) = 3.577, *p* = 0.06, *η*^2^ = 0.05
18–30 years old		
Above 31 years old		

*α* the limit of the significant level, *M* mean, *SD* standard deviation, *F(a,b)* is the variance value, *p* significant value, *η*^*2*^ effect size

### Age, the Number of “Likes”, the Importance of “Likes”, Facebook “Friends” and the Importance of Facebook “Friends” with PSMU

Table 6 illustrates participants’ responses regarding the importance of Facebook and Instagram “likes” and “friends/followers” to them.

**Table 6:** Participants’ responses per social networking site (Facebook or Instagram) on questions regarding the importance of “likes” and the importance of friends/followers (4-point scale, 1: extremely important to 4: not important at all)

	Facebook users *M* (± SD)	Instagram users *M* (± SD)
Importance of “likes”	*M*: 1.2 (± 0.44)	*M*: 2.4 (± 1.12)
Importance of “friends/followers”	*M*: 3.0 (± 0.96)	*M*: 3.5(± 0.66)

*M* mean, *SD* standard deviation

A multiple regression was conducted to investigate whether age and Facebook usage (the number of Facebook “friends”, importance of Facebook “friends”, the number of “likes”, and the importance of “likes”) are associated with PSMU. The regression model predicted 15% of the variance in PSMU, *ΔR*2 = 0.21, *F*(5, 61) = 3.24, *p* < 0.05. There was a significant negative association between the importance of “likes” and PSMU (*β* =  −1.39, *p* < 0.05), whilst none of the other variables–age (*β* =  −0.46, *p* = 0.40), the number of Facebook friends (*β* = 0.36, *p* = 0.28), the importance of Facebook friends (*β* =  −0.44, *p* = 0.39), and the number of “likes” (*β* = 1.35, *p* = 0.18)–was significant predictors of PSMU for Facebook users.

### Age, the Number of “Likes”, the Importance of “Likes”, Instagram “Followers”, and the Importance of Instagram “Followers” with PSMU

A multiple regression was conducted to study whether age and Instagram usage (the number of Instagram followers, the numbers of “likes”, the importance of Instagram friends, and the importance of “likes”) are associated with PSMU. The regression model predicts 28% of the variance in overall PSMU, *ΔR*^2^ = 0.34, *F*(5, 59) = 6.04, *p* < 0.05. There was a significant negative association between age (*β* =  −2.52, *p* < 0.05) and the importance of Instagram followers (*β* =  −3.02, *p* < 0.05) with PSMU, whilst none of the other variables–the number of Instagram followers (*β* =  −0.38, *p* = 0.49), the number of “likes” (*β* = 0.85, *p* = 0.08), and the importance of “likes” (*β* = 0.18, *p* = 0.85)–was significant predictors of PSMU for Instagram users.

## Discussion

This study investigated whether social media (Facebook and Instagram) problematic use and individuals’ well-being (self-esteem, depression, and loneliness) differed and associated with the participants’ age, the number and importance of received “likes”, and the number and the importance of social media “friends/followers”. It was expected not only to explore the relationship between the two social media platforms but also to gain more in-depth details about the association of Instagram usage and psychological well-being, as for this social media platform only limited studies have been conducted in relation to loneliness, depressive symptoms, self-esteem, and PSMU at the time that this study was conducted.

Based on participants’ responses, there was a significant difference between Facebook and Instagram users in problematic use (PSMU). Consistent with literature, this study confirmed that PSMU might be a coping mechanism for everyday difficulties, without controlling the negative consequences in daily life (Spada et al., 2014; Shensa et al., 2017). However, the various social media functionalities are differently associated with PSMU. For example, it was found that only the importance of “likes” was negatively associated to Facebook usage, whilst age and the importance of “followers” were negatively related to problematic Instagram use. The number of Facebook “friends” was also significantly positively associated with depressive symptoms, which reflected a stark contrast in direction from previous studies (Park et al., 2013; McCloskey et al., 2015). A potential explanation could be regarding the difference in the functionalities of these social media platforms and different usage. Instagram users enjoyed looking at different pictures/images, but it is Facebook users who did not use this platform just to pass their time searching for funny content. However, these users aimed to expand their friendships with others online through Facebook (Antheunis et al., 2012), which might elicit the negative feeling of depression.

Previous studies also suggested that social media “friends/followers” could enhance self-esteem (Stefanita et al., 2018); however, the findings of this study illustrate that the number of “friends/followers” for both platforms was unable to change participants’ self-esteem. Lup et al. (2015) argued that the impact of social media on one’s level of self-esteem is related to the difference between the feedback that users received with what they expected to receive, and it is not related to the number of Instagram “followers”. Metzler and Scheithauer (2017) also argued that self-esteem is negatively related to the frequency of receiving positive feedback, rather than overall Facebook use. This point also explains the finding of the negative association between “likes” and self-esteem as the number of “likes” is also perceived by individuals as received feedback (positive reward) (Burrow & Rainone, 2017). Previous studies have already presented mixed findings on this area (Stefanita et al., 2018; Marengo et al., 2021), but this study examines the number of “likes” and “friends/followers” for both platforms. This finding contradicts key theories, such as the sociometer theory of deriving self-esteem through social reinforcement (Leary, 2012), indicating that more “likes” might be related to lower self-esteem. Online social approval (i.e., “likes”) appears to have opposing negative repercussions for self-esteem, as opposed to offline social approval. Perhaps an individual’s self-esteem is increased only if they believe “likes” are reflective social validation from important offline friends (Scissors et al., 2016) and not from online “friends/followers” only. Further research on this area might reveal more details regarding individuals’ self-esteem in relation to the received “likes” from social media “friends/followers” with network density (the proportion of ties present in the network, relative to all possible ties).

Neither the number of social media “friends/followers” nor social media “likes” were significant predictors of loneliness in this study, suggesting that Facebook and Instagram users may be very selective on their online social media networks, and the reason for using social media to maintain social satisfaction. Although a previous study has found a relation between individuals’ engagement with a social media platform (i.e., Instagram browsing, broadcasting) with loneliness (Yang, 2016), the current study has not identified such association. The participants of this study have been asked to identify which of the two social media platforms they have used more, and it seems that loneliness is not associated with their selection. This may be also supported by a recent study on the use of Facebook and loneliness, which discusses the importance of active or passive use of Facebook and individuals’ characteristics in relation to the feelings of social connections to others (Brown et al., 2021).

Whilst findings give insight into how Facebook and Instagram usage and their problematic use can impact individual’s well-being, the current study has some limitations. For example, this is only a snapshot of Facebook and Instagram use, of a small sample, within a short period of time, using online questionnaire to collect participants’ responses. Future research could replicate this study utilising a longitudinal design, as this could enable insight into whether the social media platforms are influencing wellbeing, or whether well-being levels are causing engagement with social media platforms. Despite self-report measures possessing good psychometric properties, they increase the probability of social desirability and recall bias. Future research could build on the present study by utilising both objective measures of social media use (e.g., installing tracking apps onto mobile devices) and self-report measures. This could provide an interesting variation to the present study by enabling researchers to attest whether differences in well-being vary between actual time spent on social media versus perceived time spent on a particular platform. Lastly, the sample only included participants over 18, whilst the minimum required age for individuals to create a social media account is 13. Thus, these findings are limited in generalisability towards younger adolescents. Future researchers could expand the sample to include this age demographic. Furthermore, future research could investigate whether PSMU and well-being vary between age groups and social media type, as previous research suggests that younger adolescents experience more detrimental effects of social media (Richards et al., 2015). Even though this study controlled for age difference in problematic use and well-being, the social and demographic differences between Facebook and Instagram Users and their motivations should be further explored. Finally, future research should move away from solely investigating Facebook and Instagram. Although such social media platforms are highly popular, significant associations with well-being might be established by including a broader range of diverse platforms (e.g., LinkedIn, Twitter, Snapchat) (Donnelly & Kuss, 2016).

From these findings, there are several implications to be considered. An important one is that PSMU may have a detrimental effect on peoples’ lives. Thus, clinicians could be advised to specifically focus these individuals’ sessions on techniques to manage and attenuate PSMU (for example, by advising clients to set an enforced timer on their phones or implement distraction techniques when excessive preoccupation with Facebook or Instagram occurs). This insight could be beneficial for wellbeing organisations and clinicians aiming to advise individuals on effective methods for improving well-being. Secondly, it should be acknowledged that Facebook friends and likes could be particularly detrimental for well-being, in particular self-esteem and depressive symptoms. Promotion of offline social interactions should be advised in well-being centres for those presenting a high level of social media use. Furthermore, individuals could be encouraged to increase their self-esteem through alternative means (i.e., voluntary work), as opposed to receiving “likes” online. Finally, well-being may not be social media platform dependent. This means that future campaigns designed to improve well-being should not be tailored towards a particular platform.

Overall, this study has discussed that the individual’s well-being may not dramatically differ between Facebook and Instagram use, contributing to the limited research studies about the problematic use of Instagram. One common attribute of Facebook and Instagram, such as “likes” and PSMU, may be common predictors of well-being that influence both Facebook and Instagram users alike. The findings of this study further reinforce that the relationship between individual’s well-being and social media is complex. The current study may provide future researchers a springboard on this topic, allowing them to reconsider whether PSMU and social media type alone is sufficient explanatory factors for differences in well-being.

## Footnotes

1 The regression model focuses on the relationship between a dependent variable and a set of independent variables. The dependent variable is the outcome, which is expected to be predicted, using one or more independent variables. The *x*% of the variance in scores equals the correlation coefficient *R*^2^, and it represents how far the observed values differ from the average of predicted values.
